# Hepatitis B and C infection in haemodialysis patients in Libya: prevalence, incidence and risk factors

**DOI:** 10.1186/1471-2334-12-265

**Published:** 2012-10-20

**Authors:** Wiam A Alashek, Christopher W McIntyre, Maarten W Taal

**Affiliations:** 1 School of Graduate Entry Medicine, University of Nottingham, Nottingham, UK; 2 Department of Renal Medicine, Derby Hospitals NHS Foundation Trust, Derby, UK

**Keywords:** Haemodialysis, Hepatitis B, Hepatitis C, Incidence, Libya, Nosocomial infection, Prevalence

## Abstract

**Background:**

Patients receiving maintenance haemodialysis (HD) are at higher risk for acquiring Hepatitis B Virus (HBV) and Hepatitis C Virus (HCV) infections than the general population. Strict infection control measures are essential to prevent nosocomial transmission. We aimed to investigate the incidence and prevalence of HBV and HCV infection in the HD population of Libya as well as risk factors for infection.

**Methods:**

All adult patients receiving maintenance HD (n=2382) in Libyan dialysis centres (n=39) were studied between May 2009 and October 2010. Testing for Hepatitis B surface antigen (HBsAg) and anti-HCV antibodies was performed at initiation of dialysis and every 3–6 months thereafter. Patients who were sero-negative for HBV and HCV (n=1160) were followed up for 1 year to detect sero-conversions.

**Results:**

Participant median age was 49 years and 58% were male. 831 patients (34.9%) were sero-positive for HBV and/or HCV (anti-HCV positive 31.1%; HBsAg positive 2.6%; both positive 1.2%). Of the sero-positive patients 4.7% were known to be infected before the initiation of HD. The prevalence of HBV±HCV infection varied widely between HD centres from 0% to 75.9%. Sero-positive patients were younger, had longer time on dialysis and more previous blood transfusions. Prospective follow-up revealed an incidence of sero-conversion of 7.7% during 1 year (7.1% HCV; 0.6% HBV). Wide variation in rates of newly acquired infections was observed between dialysis centres. All new HBV cases were referred from centres already treating HBV infected patients. New HCV infections were reported in most centres but the rate of HCV sero-conversion varied widely from 1.5% to 31%. Duration of dialysis, history of previous renal transplant and history of receiving HD in another centre in Libya were significantly associated with sero-conversion.

**Conclusion:**

Patients on maintenance HD in Libya have a high incidence and prevalence of HCV infection and lower rates of HBV infection. The factors associated with HBV and HCV infection are highly suggestive of nosocomial transmission within HD units. Urgent action is required to improve infection control measures in HD centres and to reduce dependence on blood transfusions for the treatment of anaemia.

## Background

Chronic infections with Hepatitis B Virus (HBV) and Hepatitis C Virus (HCV) are associated with serious health risks due to hepatic cirrhosis and hepatocellular carcinoma. Patients receiving maintenance haemodialysis (HD) therapy are at increased risk for acquiring these infections and have a higher prevalence of HBV and HCV than the general population [1,2]. Prior to effective screening of blood donations, HCV infection was associated with blood transfusions needed to correct the anaemia associated with kidney disease [3,4] but patient to patient transmission in HD units is also reported [5,6]. HBV infection is usually due to patient to patient transmission within HD units [7]. Recognition of the risk of nosocomial infection has resulted in recommendations that strict infection control procedures should be followed on HD units; patients with blood-bourne virus infections should be isolated from sero-negative patients during dialysis and patients as well as staff should be vaccinated against hepatitis B [8,9]. The introduction of blood donor screening and a reduction in blood transfusions due to the availability of recombinant erythropoietin has significantly reduced the incidence of new HCV infections among HD patients in many countries [10-12].

Libya provides free access to maintenance HD for end stage kidney disease through a rapidly expanding network of centres. Although there are no national dialysis practice guidelines or infection control polices enforced by health care authorities, there is general agreement that patients on HD should be screened for HBV and HCV infection before the initiation of HD and monitored every 3–6 months thereafter [13]. Sero-positive patients are dialysed on dedicated machines either in an isolated area or alongside sero-negative patients if space does not allow isolation [13]. A national serological survey for HBV and HCV infections among the general population was performed in Libya during 2003 and revealed prevalences of 2.2% and 1.2% for HBV and HCV, respectively [14]. Other local surveys reported that the rate of HBsAg positivity among blood donors ranged from 1.3% to 4.6% [15], while the rate of HCV antibodies was 1.2% [16]. Global data indicate that the prevalence of HBV and HCV infection is high in populations of Africa and the Middle Eastern regions [17-19]. HCV infection was estimated by World Health Organisation to affect 4.6% of the Eastern Mediterranean population and 5.3% of the population of Africa [20].This study aimed to investigate for the first time the incidence and prevalence of HBV and HCV infection in the entire HD population of Libya.

## Results

The median age of adult HD patients included was 49 years (range 36–61 years) and 58% were male. A total of 831 patients (34.9%) were sero-positive for HBV and/or HCV. The majority of infected patients were positive for anti-HCV antibodies (31.1%) and 2.6% were HBsAg positive. Twenty-eight patients (1.2%) had mixed infection with HBV and HCV (Additional file 1: Table S1). Of the sero-positive patients 4.7% were known to be infected before the initiation of HD. Overall the prevalence of sero-positivity was similar among males and females (35.8% versus 33.7%, respectively) but males comprised a greater proportion of those with HBV or combined infection (Additional file 1: Table S1; P=0.01 for comparison between groups). Hepatitis B vaccine had been administered in 1216 of 1520 patients but antibody levels were not checked post vaccination. Vaccination status was not documented in 862 patients.

**Figure 1:**
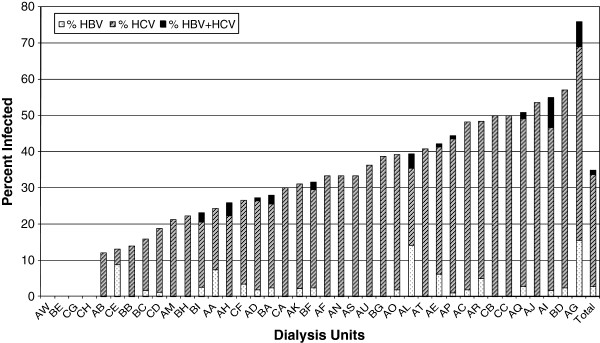
Prevalence of HBV and/or HCV sero-positivity in different haemodialysis centres in Libya.

The prevalence of HBV and/or HCV infection varied widely between HD centres from 0% to 75.9%. Four centres had no sero-positive patients and half of centres were free from HBV infection. Patients sero-positive for both viruses were found in 28.2% of centres (Figure 1).

Univariate analysis of potential risk factors for infection with HBV and/or HCV was performed by comparing infected and non-infected patients (Table 1). Sero-positive patients were younger and had been receiving dialysis for substantially longer. History and number of blood transfusions was also significantly associated with sero-positivity. Mean values for Alanine Aminotransferase and Aspartate Aminotransferase were higher in sero-positive patients despite being within the normal range.

**Table 1:** Factors potentially associated with HBV and/or HCV infection in haemodialysis centres in Libya

**Factors potentially associated with HBV±HCV infection**	**Sero-positive N=831**		**Sero-negative N=1551**		**P- value**
	Number*	**Value**	Number*	**Value**	
Age in years (mean± SD)	831	47.1±14.4	1551	49.5±16.2	<0.001
Males (number and percent)	831	494 (59.4%)	1551	887 (57.2%)	0.296
Whites (number and percent)	805	718 (89.2%)	1528	1312 (85.9%)	0.023
Dialysis vintage in years (median and IQR)	831	6 (3–10)	1551	2 (0.1-3)	<0.001
Previous blood transfusion (number and percent)	597	482 (80.7%)	1010	688 (68.1%)	<0.001
Number of blood transfusions (median and IQR)	597	2 (1–3)	1010	1 (0–3)	<0.001
Previous renal transplant (number and percent)	822	115 (14.0%)	1533	41 (2.7%)	<0.001
Previous dialysis abroad (number and percent)	617	376 (60.9%)	1025	540 (52.7%)	0.001
Previous dialysis in another Libyan centre (number and percent)	612	324 (52.9%)	1018	447 (43.9%)	<0.001
Haemoglobin level in g/dl (mean± SD)	831	10.2±1.9	1551	9.7±1.8	<0.001
Alanine Aminotransferase in IU/L (mean± SD)	269	27.8±20.2	353	19.6±17.9	<0.001
Aspartate Aminotransferase in IU/L (mean± SD)	232	29.6±21.1	328	21.3±20.3	<0.001
Diabetes (number and percent)	831	195 (23.5%)	1551	551 (35.5%)	<0.001
Erythropoietin treatment (number and percent)	604	450 (74.5%)	1059	830 (78.4%)	0.071

*Number of patients with valid data for each variable.

Results of the prospective study showed that 89 of 1160 previously sero-negative patients sero-converted during 1 year (incidence 7.7%). The majority (82 patients) developed anti-HCV antibodies (incidence 7.1%). Seven patients became positive for HBsAg (incidence 0.6%). Of these one had previously been vaccinated against hepatitis B, four had not been vaccinated and in two vaccination status was not documented. Age and gender distribution of those who sero-converted is shown in Additional file 2: Table S2.

**Figure 2:**
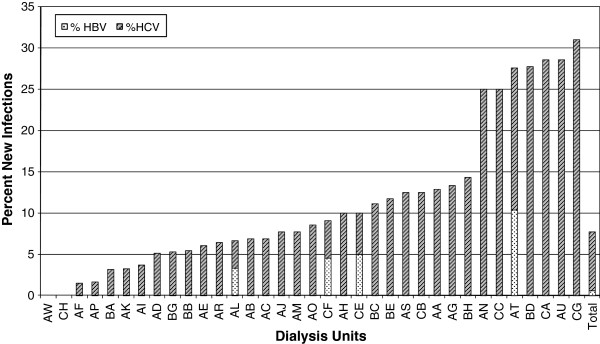
Incidence of new HBV or HCV infections in haemodialysis patients during a 1-year follow up period.

Figure 2 shows wide variation in rates of newly acquired infections between different dialysis centres. No sero-conversion was found in two small-capacity centres (one treating 2 patients and the other, 5 patients). New HBsAg positive cases were detected in 4 centres in 3.3-10.3% of previously negative patients. All new HBV cases were referred from centres already treating HBV infected patients. New HCV infections were reported in most centres (33 of 35) but the rate of HCV sero-conversion varied widely from 1.5% to 31% of patients. Most of the centres with HCV sero-conversions (31/33) were providing HD treatment to previously HCV-infected patients. However, anti-HCV antibodies were discovered in 9 patients (31%) in a centre which was previously treating exclusively sero-negative patients (coded CG). Two other HCV sero-conversions occurred in another previously sero-negative HD centre (coded BE).

There were no correlations between number of HD patients treated in each centre and the prevalence or incidence of sero-positivity to HBV or HCV. There was no difference in prevalence or incidence of HBV or HCV infection between units that: routinely practiced isolation of sero-positive patients versus those that did not; units with hand washing facilities in each cubicle versus not and units that had adequate facilities for sharps disposal versus not.

Analysis of possible risk factors for new HBV or HCV infections is shown in Table 2. Only duration of dialysis, history of previous renal transplant and history of receiving HD in another centre in Libya were significantly different between patients who sero-converted and those who remained sero-negative.

**Table 2:** Factors potentially associated with new HBV or HCV infection in HD centres

**Factors potentially associated with new HBV±HCV infection**	**Sero-converted**		**Stayed sero-negative**		**P- value**
	**N=89**		**N=1071**		
	Number*	**Value**	Number*	**Value**	
Age in years (mean± SD)	89	50.9±15.8	1071	50±16.6	0.635
Males (number and percent)	89	54(60.7%)	1071	613(57.2%)	0.528
Whites (number and percent)	89	75 (84.3%)	1056	927 (87.8%)	0.335
Dialysis vintage in years (mean± SD)	89	4.1±4.4	1071	2.2±2.6	0.001
Previous blood transfusion (number and percent)	45	34 (75.6%)	716	481(67.2%)	0.244
Number of blood transfusions (mean± SD)	45	3.6±6.9	716	1.8±2.6	0.083
Previous renal transplant (number and percent)	89	6 (6.7%)	1064	28 (2.6%)	0.028
Previous dialysis abroad (number and percent)	48	27 (56.3%)	714	395 (55.3%)	0.900
Previous dialysis in another Libyan centre (number and percent)	48	31 (64.6%)	707	371(52.5%)	0.023
Haemoglobin level in g/dl (mean± SD)	89	9.9±2.1	1071	9.6±1.8	0.220
Alanine Aminotransferase in IU/L (mean± SD)	27	21.9±22.7	274	18.6±17.8	0.272
Aspartate Aminotransferase in IU/L (mean± SD)	24	23±23.8	252	20.8±21.2	0.925
Diabetes (number and percent)	89	35 (39.3%)	1071	385 (35.9%)	0.524
Erythropoietin treatment (number and percent)	51	7 (13.7%)	729	100 (13.7%)	0.999

## Discussion

The prevalence of anti-HCV antibodies in patients receiving HD (31.1%) was remarkably high and is approximately 25 times higher than in the general population [14]. It is also higher than the prevalence of 20.5% reported by Daw et al. in a sample of 200 HD patients in 2001 in Libya [16]. Globally the prevalence of HCV among patients receiving HD varies from as low as 6.1% in Germany [21] to as high as 76% in Casablanca [22]. In general, North Africa and the Middle East are high prevalence areas both in the general population and in HD patients [20]. Previous studies from the region have reported a prevalence of anti-HCV antibodies in HD patients of 50% in Saudi Arabia [23], 42% in Tunisia [24], 20.2% in Turkey [25] and 21% in Jordan [26]. In contrast, the observed prevalence of HBV infection (2.6%) is similar to the general population and similar to that reported in HD patients in other regions including Europe (4.1%), Japan (2.2%) and the USA (2.4%) [27]. A study sample from the Dialysis Outcome and Practice Patterns Study that included 8615 adult HD patients from 308 dialysis facilities in Western Europe and the United States, reported prevalence rates for HBV infection ranging from 0% to 6.6% [28]. Studies from less developed countries estimated that the proportion of HBsAg carriers in the HD population varies from 2% to 20% [29-32]. According to the 2008 Saudi Centre for Organ Transplantation (SCOT) report, HBV sero-positivity was 4.6% in the Saudi HD population [33] while among Jordanian HD patients it was 5.9% [34]. In general, the prevalence and incidence of HBV and HCV infections in HD patients reflects the prevalence of these infections in the general population, the quality of healthcare services in a community and the standards of infection control practices in HD units. The importance of HBV and HCV as a health risk in patients on HD is illustrated by our observation that 3% of deaths in Libyan HD patients during a 1 year observation period were due to liver failure and that 13 of the 14 patients who died of liver failure were sero-positive for HCV and/or HBV [35].

Our data show that sero-positive patients were significantly younger on average than sero-negative patients. This observation is in agreement with a previous report from Libya showing that the highest prevalence of HCV antibodies was observed in HD patients aged 36–55 years [16]. Other studies [28,36,37] have reported a higher prevalence of HBV or HCV sero-positivity in older patients and the reason for this difference in not clear. On the other hand, the prevalence and incidence of HBV or HCV sero-positivity was significantly related to the length of time on HD. This is consistent with nosocomial transmission related to dialysis since longer duration of dialysis represents a longer period at risk of acquiring an infection. Similar observations have been reported by other authors [38-41]. Prevention of nosocomial transmission is of vital importance in Libya as HCV antiviral treatment is expensive and its availability is limited to only a few centres.

A positive history of blood transfusions as well as the number of blood transfusions was strongly associated with HBV or HCV infection at baseline, but not with new infections. Prior to the introduction of effective screening of blood donors, blood transfusions were recognised as the leading source of HCV infection and some of these infections may have been acquired before adequate screening was introduced [21,40]. In addition it is possible that some blood donors with HCV infection are being missed by current screening procedures and these may need to be reassessed [42,43]. On the other hand the lack of association between blood transfusions and new infections suggests that fewer infections are acquired by this route than previously. A large proportion of patients had previously received blood transfusions. The risk of infection could therefore be further reduced by more effective management of anaemia with iron supplementation and erythropoietin. In accordance with other studies [41,44,45], HBV or HCV infection was more prevalent in patients with a history of previous renal transplant. Infection in these patients might have been transmitted from an infected donor kidney or blood transfused peri-operatively. This observation emphasizes the need for adequate screening of potential kidney donors, which is deficient in some countries. The shortage of donated kidneys in Libya induces many patients to seek a transplant abroad.

Another concern raised by the current study is that HBV or HCV infection was associated with a history of HD in another centre either in Libya or abroad. Many patients travel for social reasons but some also transfer to a maintenance HD centre after initiating dialysis as an emergency in a specialised centre providing acute services or may travel to another centre for surgery to create an arteriovenous fistula [13]. The association of hepatitis virus infection with travel suggests that the risk of nosocomial infection varies between dialysis centres within Libya and abroad. The former is confirmed by our data showing a marked variation in both prevalence and incidence of HBV and HCV infection among Libyan HD units (Figures 1 and 2). These observations emphasize the importance of isolating patients following their return and monitoring them for sero-conversion.

Prospective follow up of sero-negative HD patients enabled us to verify 89 sero-conversions for HBV or HCV during 1 year, giving an overall incidence of 7.7% for new infections. The incidence rate of 0.6% for HBsAg sero-conversion is similar to that reported in Europe, Japan and the USA (0.4-1.8 per 100 patient-years) [28]. Three new HBsAg positive patients were detected in a single centre that was treating 20 other HBsAg positive patients and 2 new cases were detected in another centre that was treating 14 HBsAg positive patients, suggesting that nosocomial transmission probably occurred. We observed a high incidence of new HCV infections during the 1-year observation period (7.2%). The reported incidence of new HCV infections varies considerably between countries. A rate as low as 0.4% was observed in France from 1997 to 2000 [46] but higher rates have been reported from the Mediterranean region. According to the 2008 SCOT report, the annual rate of HCV sero-conversion in Saudi HD patients was 7-9% [33] while in Jordan it was 2.6% [26]. In our study most new cases were observed in centres treating other patients with HCV infection, suggesting nosocomial transmission. Interestingly 9 new HCV infections were observed in one unit and 2 in another that previously accepted only patients without HCV infection. This raises the possibility of transmission from a carrier that was not detected by current screening procedures.

A striking observation from this study is the wide variation in incidence and prevalence of HBV and HCV infections among different HD units (Figures 1 and 2). Interestingly none of the potential centre-related factors that we assessed formally explained this variation. On the other hand, we observed variations in other practices that may be relevant. Most facilities faced a problem of increasing number of patients and most of them responded by adding more HD stations at expense of space and staff. Infection control precautions also varied widely between centres. They were strictly enforced in some places but frequently breached in others. This seemed to depend on staff initiative rather than national guidelines. On the other hand, dialyser reuse was not permitted and all bloodlines as well as other consumables were disposed after a single use [13]. Some brands of HD machines were equipped with a sphygmomanometer. Otherwise, most non- disposable instruments used in HD environment were shared between sero-positive and sero-negative patients. The use of multi-dose vials of heparin was common and is likely to have been an important cause of nosocomial infections. Many patients started HD without being vaccinated against HBV. Even in vaccinated patients the antibody titre was not assessed. The wide variation in HBV and HCV prevalence and incidence between dialysis centres implies that there is potential to reduce blood-borne virus infection by transferring best practice from HD centres with low infection rates. In particular infection control procedures should be investigated in centres with high infection rates and the use of multidose heparin vials must be stopped urgently. Previous studies from the region show that with appropriate intervention HCV infection rates in HD centres may be substantially improved. For example in Iran, HCV prevalence reduced from 14.4% in 1999 to 4.5% in 2006 [47] and in Saudi Arabia from 2.4% in 2001 to 0.2% in 2005 [48].

Several limitations of this study should be conceded. Medical records were often incomplete and additional clinical information was frequently obtained by interviewing staff and patients. Serological testing was done in local laboratories and it is likely that there was some variation in the quality of testing. Data regarding hepatitis B core antibodies (HBcAb) or hepatitis B DNA were not available. In one recent study of haemodialysis patients in Egypt who were negative for HBsAg, hepatitis B DNA was detected in 4.1% and HBcAb in 20% [49]. It is therefore possible that we failed to detect cases of occult hepatitis B infection. Testing for HCV relied on a third generation ELISA to detect anti-HCV antibodies and confirmation or genotyping with PCR is currently not available in most centres.

## Conclusion

In conclusion, patients on maintenance HD in Libya have a high incidence and prevalence of HCV infection and lower rates of HBV infection. The factors associated with HBV and HCV infection are highly suggestive of nosocomial transmission within HD units. Urgent action is required to improve infection control measures in HD centres and to reduce dependence on blood transfusions for the treatment of anaemia. The data presented were obtained before the recent conflict in Libya. It is possible that disruption of services due to the conflict may have exacerbated the problem of hepatitis virus infection in HD patients.

## Methods

This descriptive study was carried out in all HD centres treating adult patients in Libya (n=39) from May 2009 to October 2010. Phase I of the study was a collection of cross-sectional data regarding all adult patients in maintenance HD facilities (n=2382). Large and medium capacity HD facilities were visited by the researchers in order to collect data. Patient records were used to obtain patients’ age, gender, time on HD, medical history, sero-positivity to HBV and HCV as well as other laboratory results. Sero-positivity to HBV was defined by detection of hepatitis B surface antigen (HBsAg) and sero-positivity to HCV by detection of anti-HCV antibodies by a third generation enzyme linked immunoassay (ELISA). ELISA tests were performed in local laboratories. In addition, 1732 patients from 24 centres were interviewed regarding other potential risk factors for HBV and HCV infection. These included history of blood transfusions and history of HD in another centre within Libya or abroad. Data was also obtained regarding infection control procedures at each HD facility. For small and remote facilities, data collection forms were posted to clinical supervisors who were requested to collect information about their patients. They were contacted frequently by phone to deal with any queries related to the required variables. Forms were returned within 30 days.

Phase II of the study was to prospectively detect sero-conversion to HBV or HCV in previously sero-negative HD patients during 1 year of follow-up. This step included a cohort of 1160 patients from 35 centres (four centres were excluded because of incomplete information). All sero-conversions were recorded and included even if patient was transplanted or died afterwards. The research team frequently monitored HD patients during the year to assure the inclusion of every new sero-conversion. All new sero-conversions were retested to confirm the positive result. Field visits were repeated at the end of 1 year to validate the information.

Data are presented as mean±SD if normally distributed or median (interquartile range) if not. Analysis was performed using SPSS version 16.0. A Chi-square test was used to compare frequencies between groups. Correlations were tested with a Pearson’s test. A t-test was used to compare means between groups for data with normal distribution or Mann–Whitney test for non-parametric data

Permission to conduct the study was granted from the ministry of health. The project was approved by the Libyan National Committee for Bioethics and Bio-safety. Patients gave written informed consent to be interviewed. The study was performed by a Libyan researcher with support from a Nephrology Department in the UK.

## Competing interests

The authors have no conflicts of interest to declare. The results presented in this paper have not been published previously in whole or part, except in abstract form.

## Authors’ contributions

The contribution of each of the authors was as follows: WA: study design, collection of all data, analysis of data, writing of manuscript. CWM: study design, review of data, writing and revision of manuscript. MWT: study design, review of data, writing and revision of manuscript. All authors read and approved the final manuscript.

## Authors’ information

WA is based in Libya and conducted this study as part of a PhD project, supervised by CWM and MWT, who are consultant nephrologists in the UK.

## Pre-publication history

The pre-publication history for this paper can be accessed here:

http://www.biomedcentral.com/1471-2334/12/265/prepub

## Supplementary Material

Additional file 1 **Table S1.** Frequency, age and gender distribution of HBV and/or HCV sero-positive haemodialysis patients. Data are number (percent) or median (interquartile range). Click here for file

Additional file 2 **Table S2.** Frequency, age and gender distribution of patients who sero-converted during 1 year of follow-up. Data are number (percent) or median (interquartile range). Click here for file
