# Supplementary material for: Hepatitis B and C infection in haemodialysis patients in Libya: prevalence, incidence and risk factors
Source: BMC Infect Dis. 2012 Oct 20;12:265. doi: 10.1186/1471-2334-12-265 (PMC3507892; doi:10.1186/1471-2334-12-265)
Supplement: Additional file 1 — Table S1. Frequency, age and gender distribution of HBV and/or HCV sero-positive haemodialysis patients. Data are number (percent) or median (interquartile range). [file 1471-2334-12-265-S1.doc]

Supplementary Table 1. Frequency, age and gender distribution of HBV and/or HCV sero-positive haemodialysis patients. Data are number (percent) or median (interquartile range).

|  | | HBV  n=62 | HCV  n=741 | HBV+HCV  n=28 | Total  n=831 |
| --- | --- | --- | --- | --- | --- |
| Frequency | Male | 45 (72.6%) | 427 (57.6%) | 22 (78.6%) | 494 (59%) |
|  | Female | 17 (27.4%) | 314 (42.4%) | 6 (21.4%) | 337 (41%) |
| Age (years) | Male | 49 (33-58) | 46 (36-59) | 40 (31-45) | 46 (36-59) |
|  | Female | 44 (33-61) | 48 (34-59) | 44 (42-54) | 47 (35-59) |
