# Supplementary material for: Hepatitis B and C infection in haemodialysis patients in Libya: prevalence, incidence and risk factors
Source: BMC Infect Dis. 2012 Oct 20;12:265. doi: 10.1186/1471-2334-12-265 (PMC3507892; doi:10.1186/1471-2334-12-265)
Supplement: Additional file 2 — Table S2. Frequency, age and gender distribution of patients who sero-converted during 1 year of follow-up. Data are number (percent) or median (interquartile range). [file 1471-2334-12-265-S2.doc]

Supplementary Table 2. Frequency, age and gender distribution of patients who sero-converted during 1 year of follow-up. Data are number (percent) or median (interquartile range).

|  | | HBV  n=7 | HCV  n=82 | Total  n=89 |
| --- | --- | --- | --- | --- |
| Frequency | Male | 5 (71.4%) | 49 (59.8%) | 54 (61%) |
|  | Female | 2 (28.6%) | 33 (40.2%) | 35 (39%) |
| Age (years) | Male | 50 (35-67) | 50 (38-64) | 50 (38-64) |
|  | Female | 57 (40-73) | 52 (44-64) | 52 (43-65) |
